# Evaluation of Colon Capsule Utilisation in Europe—CAPTURE EU Survey Findings

**DOI:** 10.3390/jcm14010099

**Published:** 2024-12-27

**Authors:** Ian Io Lei, Alexander Robertson, Anastasios Koulaouzidis, Ramesh Arasaradnam, the international Capsule Endoscopy Research (iCARE) Group

**Affiliations:** 1 Institute of Precision Diagnostics & Translational Medicine, University Hospital of Coventry and Warwickshire, Clifford Bridge Rd, Coventry CV2 2DX, UK; r.arasaradnam@warwick.ac.uk; 2 Department of Digestive Diseases, University Hospitals of Leicester NHS Trust, Leicester LE1 7RH, UK; alexander.robertson7@nhs.net; 3 Surgical Research Unit, Odense University Hospital, 5700 Svendborg, Denmark; akoulaouzidis@hotmail.com; 4 Department of Clinical Research, University of Southern Denmark, 5230 Odense, Denmark; 5 Department of Gastroenterology, Pomeranian Medical University, 70-204 Szczecin, Poland; 6 Department of Medicine, OUH Svendborg Sygehus, 5700 Svendborg, Denmark; 7 Warwick Clinical Trials Unit, University of Warwick, Coventry CV4 7AL, UK; 8 Warwick Medical School, University of Warwick, Coventry CV4 7AL, UK; 9 Leicester Cancer Centre, University of Leicester, Leicester LE1 7RH, UK

**Keywords:** colon capsule endoscopy (CCE), panenteric capsule endoscopy, colonoscopy, capsule endoscopy, colon cancer, colorectal cancer, incomplete colonoscopy, inflammatory bowel disease

## Abstract

**Background/Objectives:** Colon capsule endoscopy (CCE) is a non-invasive method for visualising the colon, but its clinical adoption has been slow. Although the COVID-19 pandemic reignited interest in CCE, its role in conventional gastrointestinal investigations remains unclear, leading to varied practices across Europe. This highlights the need for a comprehensive understanding of diverse approaches to CCE in clinical practice. **Method:** A web-based survey was conducted from January to July 2024, targeting European gastroenterologists and colorectal surgeons interested in capsule endoscopy through the International Capsule Endoscopy Research (iCARE) Group. The survey aimed to understand CCE application across Europe and investigate factors influencing its uptake. **Results:** Thirty-eight (n = 38) valid responses were received from 19 European countries. While 88% reported access to CCE, only 45% had local services readily available, and just 7% included CCE in national guidelines. The most common indication for CCE was for patients who declined or could not tolerate colonoscopy (30%), with 77% of CE specialists preferring its use in fit patients. Ease of access was significantly associated with service availability (*p* = 0.0358). Barriers to uptake included lack of reimbursement, insufficient knowledge, and limited use in research settings. Only 27% of specialists viewed CCE positively, while 57% had a negative perception. **Conclusions:** This study reveals the wide variation in CCE practices and critical factors influencing its uptake. Understanding common indications and patient groups is the key to guiding its future development, particularly as AI and telemedicine enhance its potential for rapid full digestive tract visualisation.

## 1. Introduction

Colon capsule endoscopy (CCE) provides a new way to endoscopically visualise the colonic lumen, serving as an alternative to traditional colonoscopy and computed tomography (CT) colonography (CTC). Its diagnostic accuracy has been established in the literature, particularly for detecting polypoidal lesions [1,2,3,4,5] and assessing inflammatory bowel disease [6,7], demonstrating comparable performance to colonoscopy. Despite relevant guidelines, several meta-analyses [2,4,8], and increasing availability, clinical indications for CCE and post-CCE follow-up procedures remain an issue of continuous clinical research. The ESGE 2012 guideline suggested that CCE could help increase colorectal cancer (CRC) screening participation because it is non-invasive, does not require sedation, and does not involve gas insufflation [9]. Despite these aspirations, though, its broader adoption has been rather slow [10].

The COVID-19 pandemic renewed interest in CCE, particularly in Scotland and England, where healthcare systems sought alternatives to in-person procedures. Despite recent updates on other imaging modalities, such as the CTC 2020 guideline, the precise role of CCE within the existing diagnostic frameworks of conventional colonoscopy and CTC remains unclear. Earlier guidelines recommended CCE primarily for average-risk individuals, while high-risk patients were advised to undergo direct colonoscopy, except in cases where colonoscopy was incomplete or not feasible. In such cases, same-day CCE was preferred. However, the lack of robust evidence for symptomatic patients has led to unfavourable recommendations for CCE use in this cohort, as noted in the latest updated 2020 ESGE guidelines [11].

Similarly, CCE is not recommended as a first-line tool for CRC screening in the revised guidelines. Furthermore, insufficient evidence supports its routine use in the diagnostic work-up or surveillance of patients with suspected or known inflammatory bowel disease (IBD). However, it may have the potential for monitoring inflammation in ulcerative colitis [9]. For post-polypectomy surveillance, the evidence also remains inadequate to recommend CCE.

Although various studies and guidelines have examined CCE’s recommended uses, significant differences exist across European countries. For example, the NHS England pilot project included symptomatic patients with a quantitative faecal immunochemical test (qFIT) level of less than 100 μg/g and post-polypectomy surveillance patients. The project aimed to reduce the need for subsequent colonoscopy after CCE and improve its cost-effectiveness nationally [12]. In contrast, the ScotCap study in Scotland included a higher qFIT threshold at 250 μg/g and other additional indications, such as surveillance of patients with a personal or family history of CRC, colonic polyposis histology, and hereditary non-polyposis colorectal cancer (HNPCC), emphasising CCE’s scalability and diagnostic speed [13]. This highlights significant regional disparities even within the UK.

Different countries prioritise different patient cohorts for CCE, and the unclear evidence surrounding its indications complicates its integration into clinical practice. Therefore, a comprehensive understanding of the diverse approaches in different European countries and patient cohorts, supported by existing evidence, may facilitate the development of a more uniform or standardised approach to using CCE in clinical practice.

Aim:

To outline approaches to CCE application across various healthcare systems in European countries. We will provide a summary of the key findings. This should then provide some insights for developing optimal strategies and indications for CCE use.

## 2. Methods

### 2.1. Survey Design

**Figure 1:**
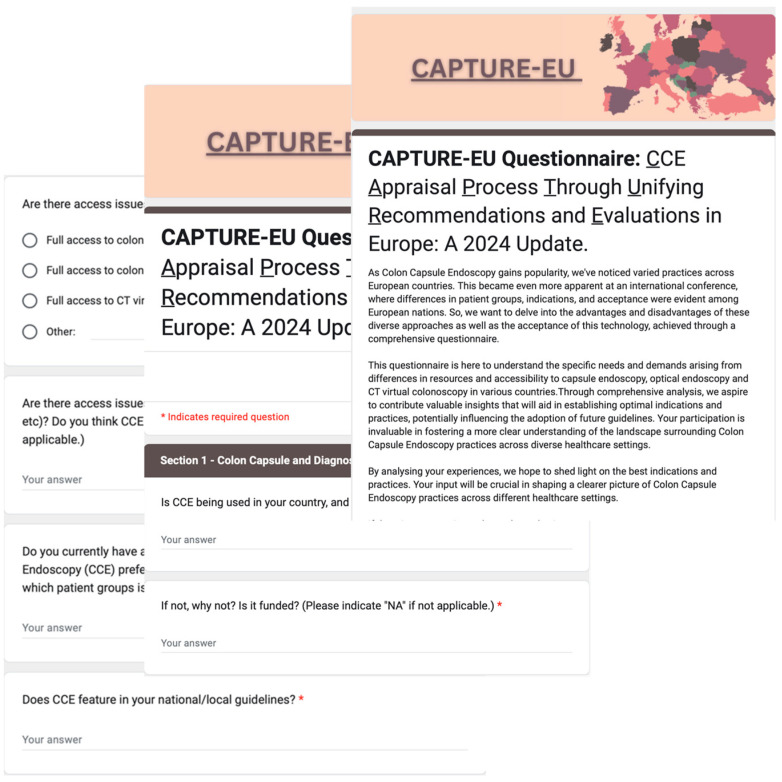
The visual representation of the online survey.

The CCE Appraisal Process Through Unifying Recommendations and Evaluations in Europe (CAPTURE EU) is a web-based survey using Google Forms (Google, Mountain View, CA, USA). This cloud-based tool allows for the creation, distribution, circulation, and analysis of the survey forms. Targeting capsule endoscopy specialists across Europe, the survey was available in English and accessible online at https://forms.gle/sgzigo4gUAfQyP4p9 (accessed on 10 January 2024). The survey was designed by the study’s steering group members, A.R. and I.L, with pilot testing performed by A.K., a seasoned CCE expert with over 5000 CCE reads. Feedback from the pilot test was received with subsequent incorporation into the final version of the survey. It consists of 20 items structured into six sections, as detailed in Table 1 and Figure 1.

### 2.2. Study Cohort and Survey Distribution

This study was aimed at specialists defined as gastroenterologists or surgeons with experience in capsule endoscopy or CCE within their respective European countries. All participating specialists provided consent to participate in this research survey. The initial aim was to include 44 specialists, one from each European country, with CE experience.

Between 9 January 2024 and 10 July 2024, the target group was contacted via email invitations and International Capsule Endoscopy Research (iCARE) Group professional society announcements. A 4-weekly email reminder was sent via email to improve uptake. Participants were also invited to invite colleagues from other European countries who still need to participate or to forward their contact information to the study team for follow-up. Participants could participate in the survey only once and withdraw from the study anytime by closing the browser. All responses remain confidential, and each was equally weighted when calculating the survey completion rate. The online survey featured auto-save functionality, a part of the Google Forms feature (https://www.google.com/forms/about/, (accessed on 10 January 2024)), and only completed responses were considered valid.

### 2.3. Outcome

The primary outcome is understanding the different aspects of CCE application in various European countries to summarise the approaches, protocols, and clinical practice, thereby identifying best practices, regional variations, and potential areas for standardisation and improvement. The secondary outcome includes investigating factors influencing the uptake of CCE service in different countries, such as the availability of colonoscopy and CTC, funding, national guidelines, and clinicians’ and patients’ perceptions of CCE.

### 2.4. Statistical Analysis

The statistical analysis was predominantly performed using Microsoft Excel [Microsoft 365, (Washington, DC, USA)] and R software version 3.6.0 [R Foundation for Statistical Computing, Vienna, Austria] [14]. All the categorical variables were expressed as percentages. Pearson’s χ^2^ was used for categorical variables analysis. Univariable and multivariable binary logistic regression analyses were conducted to identify the factors associated with increased current CCE service availability, preference between CCE and CTC, and future CCE use. A *p*-value of less than 0.05 was considered statistically significant.

## 3. Results

### 3.1. The Participants’ Geographic Demographics and Accessibility to CCE

We received 43 responses, five of which were excluded due to there being one duplicate response and four from countries outside the EU. This resulted in 38 valid responses from 19 European countries (Table 2). No responses were received from the remaining 25 European countries. All the participants are gastrointestinal (GI) clinicians with a special interest in CE. The further breakdown of various CCE indications across different EU countries is included in Table A3 in the Appendix A.

**Figure 2:**
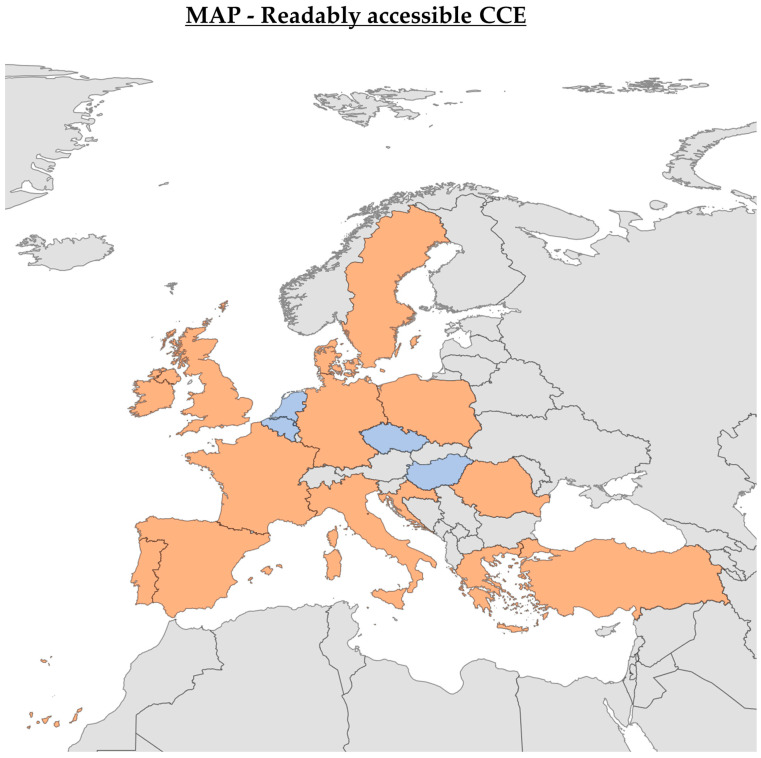
CCE Accessibility in Europe—The map illustrates the availability of colon capsule endoscopy (CCE) across European countries. Orange indicates countries where CCE is available, blue shows countries where it is unavailable, and grey represents countries with unknown CCE availability status. The diagram was created using Bing Maps, with support from Australian Bureau of Statistics, GeoNames, Microsoft, Navinfo, Open Places, OpenStreetMap, Overture Maps Foundation, TomTom, and Zenrin [15].

**Figure 3:**
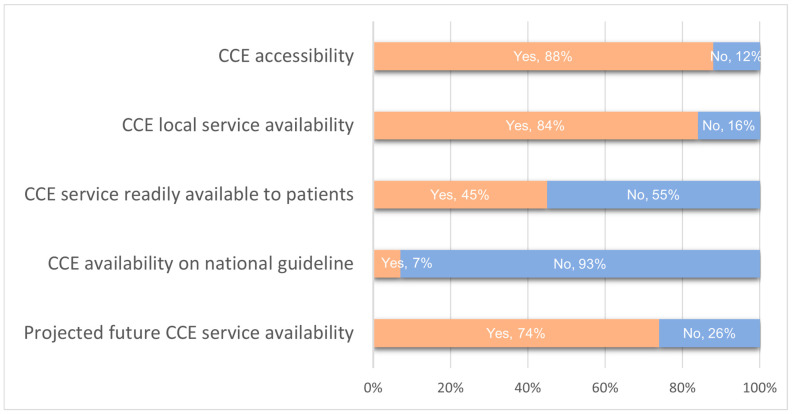
Overview of CCE accessibility and service parameters.

Accessibility to CCE was reported at 88%, with 84% having CCE services available (see Figure 2). However, only 45% had readily accessible local CCE services for patients. Moreover, just 7% reported having national guidelines that include CCE (Figure 3).

### 3.2. Issues with GI Investigations Access

**Figure 4:**
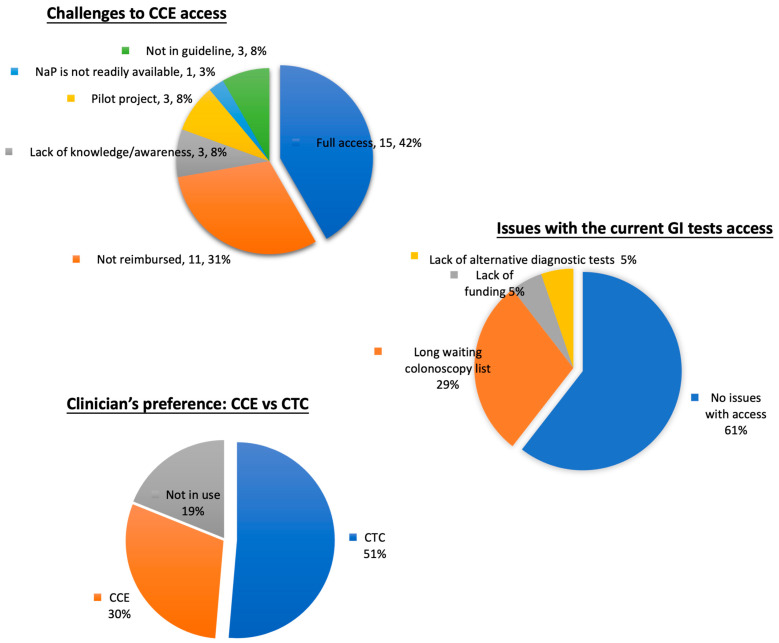
Charts of challenges to CCE access, issues with current GI test accessibility, and clinicians’ preferences between CCE and CTC.

Only 39% of specialists from 11 countries reported issues with access to lower GI investigations. Of these, 29% attributed the problem to long waiting colonoscopy lists, while other causes included a lack of public funding (5%) and the absence of alternative diagnostic tests (5%). Regarding challenges to CCE access, 58% reported difficulties accessing CCE, with 31% stated that a lack of government reimbursement was the primary issue. Other significant factors included a need for more awareness, limited research settings, and the absence of inclusion in national guidelines. When comparing CCE and CTC, 51% preferred CTC, while only 30% opted for CCE. Some 19% could not comment due to a lack of exposure to CCE (see Figure 4).

### 3.3. Indications, Patient Age, and Perceptions in Different Countries

There are noteworthy variations in CCE practices across different countries, with each nation having distinct indications and target patient cohorts. For instance, based on the survey findings, France and Turkey exclusively use CCE for older and frail patients, while seven other countries prefer to limit its use to fit patients only. This pattern is further corroborated by a previous French multicentre study, which primarily employed CCE in elderly patients with a mean age of 70 years as part of routine clinical practice [16]. The top three CCE indications were patients who declined or did not tolerate colonoscopy (30%), IBD surveillance and monitoring (11%), and investigations for suspected IBD (10%).

**Figure 5:**
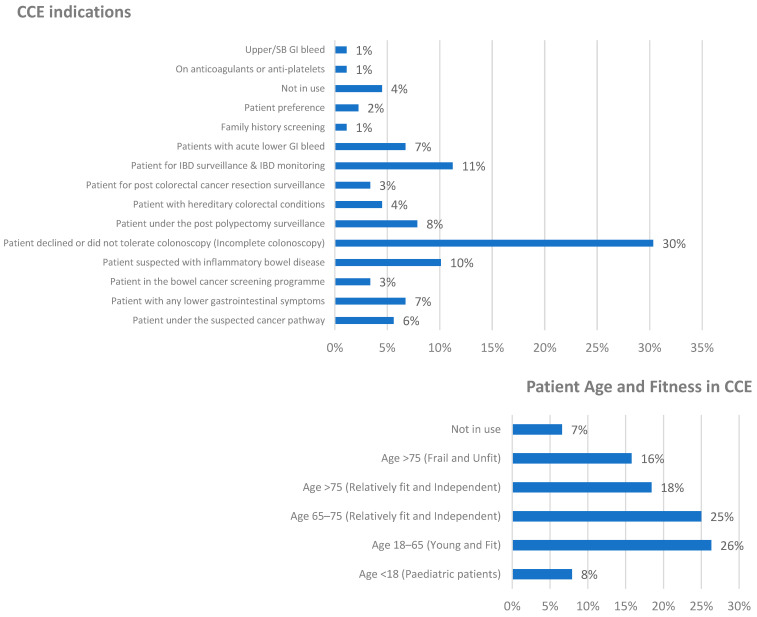
CCE indications and patient age and fitness in CCE use.

Regarding patient age, 69% of specialists prefer to perform CCE on fit patients across all age groups, with a particular preference for those aged 18–65 (26%). This age variation was also statistically significant (*p* = 0.0076) using the χ^2^ test, indicating substantial differences between these age and fitness categories among different countries (see Figure 5).

### 3.4. Diverse Perceptions of CCE

**Figure 6:**
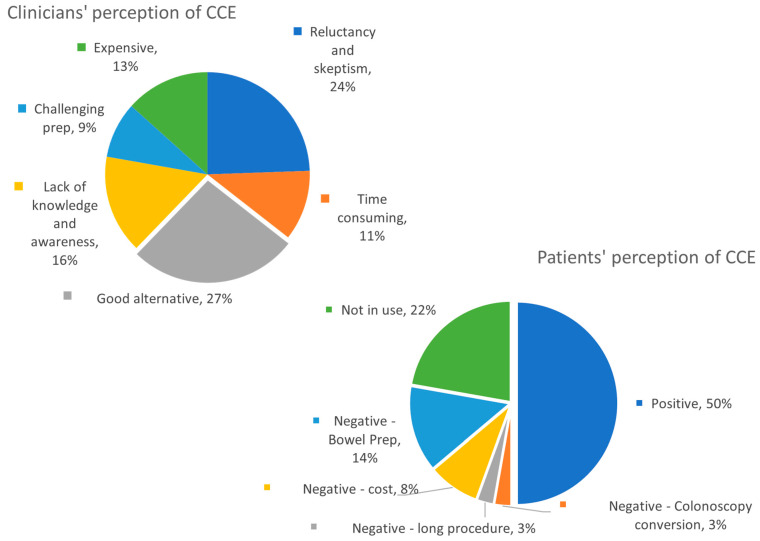
Clinicians’ and patients’ perceptions of CCE.

From the clinicians’ perspective, 27% of CCE specialists view CCE as a good alternative, while 24% perceive significant reluctance and scepticism. Other perceived issues included challenging bowel preparation, high cost, lack of knowledge and awareness, and significant time requirements for video analysis. For perceived patients’ perception of CCE, 50% viewed it positively, whereas 22% did not use CCE, and other negative perceptions included difficult bowel preparation, cost, long procedure, and the potential need for colonoscopy conversion (See Figure 6).

### 3.5. Bowel Preparation Regimen

The bowel preparation regimen for CCE is more challenging than that for colonoscopy, primarily because CCE lacks the in-vivo cleansing ability (suction and washing capabilities) available with colonoscopy. Bowel cleansing for CCE is typically evaluated using two scoring systems: the Leighton–Rex score and the CCLEAR score [17]. These systems assess the adequacy of colonic mucosal exposure in each segment, with the Leighton–Rex score also accounting for the “bubble effect,” which can impact visibility. Despite the established scoring methods and extensive experience with laxatives in colonoscopy, a universally optimal preparation regimen for CCE has yet to be determined, leading to considerable variability in protocols across centres.

**Figure 7:**
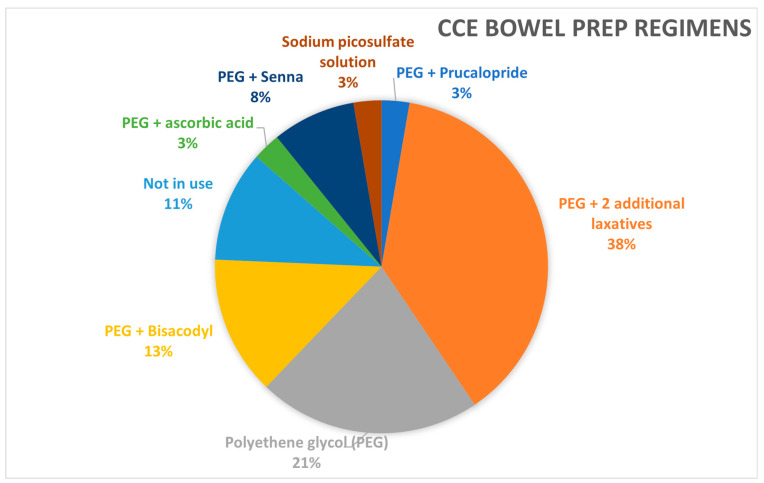
Distribution of different bowel preparation regimens.

**Figure A1:**
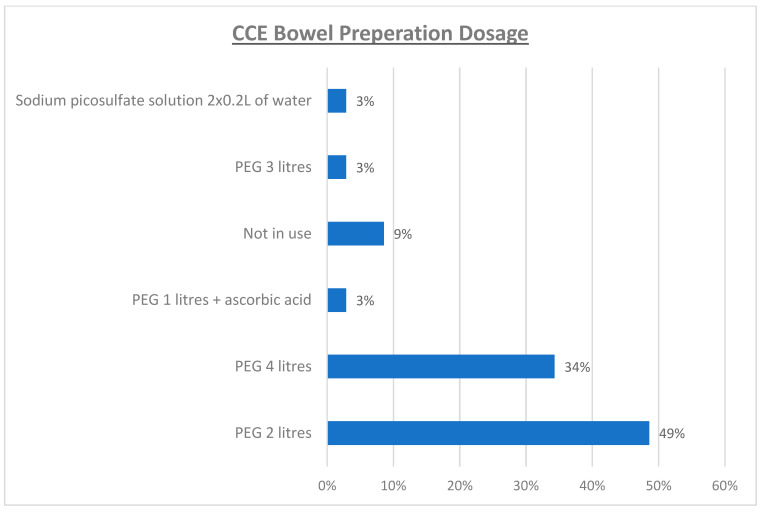
Popularity of different bowel preparation regimen dosages.

The survey identified eight distinct bowel preparation regimens across participating centres. The majority (59%) of centres use Polyethylene Glycol (PEG), either alone or in combination with two additional laxatives. A small percentage of centres (3%) utilise prucalopride specifically for bowel preparation, while 19% incorporate it into their booster regimens, particularly in Sweden, Poland, Hungary, and the United Kingdom (see Figure 7). The most commonly used dosage of PEG is 2 litres, with a variety of booster regimens detailed in the Appendix A (Figure A1).

### 3.6. Factors That Affect the Use of CCE

In our study of the factors affecting the availability of CCE services, we investigated several factors, including national CCE guidelines, preferences for CTC and CCE, perceived future CCE services, and the challenges in current GI investigations. However, we found no statistical significance after conducting univariate and multivariate logistic regression analyses (see Appendix A Table A1). Interestingly, the χ^2^ test of independence revealed a statistically significant association between the ease of accessing CCE services and the availability of local CCE services (χ^2^ test, *p* = 0.036). This finding suggests that enhancing the availability of local CCE services could be the most effective strategy to improve accessibility.

## 4. Discussion

This is the first survey in Europe to investigate the various factors that influence the availability and approach of CCE services. This study highlights significant variations in practice across different countries due to the lack of standardised indications, age group specifications, and the positioning of CCE, among other gastrointestinal investigations. Despite 88% of countries or specialists having access to CCE technology, only 45% have local CCE services readily available to their patients, indicating that this technology is not yet widely adopted.

There were several reasons for the limited uptake of CCE services. These include limited support from national guidelines, challenges accessing current GI investigations, preferences between CTC and CCE, clinician and patient perceptions, reimbursement issues, lack of awareness, and the technology primarily available for research. While no single factor showed statistical significance using the logistic regression model, the χ^2^ test indicated an association between challenges in accessing CCE and the availability of local CCE services (*p* = 0.0358), among other factors (see Appendix A Table A1). While this analysis only shows an association and not causation, it has revealed several challenges in accessing CCE: 31% of countries do not offer reimbursement; 8% lack knowledge about CCE; 93% do not include CCE in guidelines; and 8% have CCE available only through pilot research projects. These challenges collectively hinder the widespread adoption of this GI modality.

On the other hand, the problems within the current GI services should drive interest in adopting new technologies to alleviate pressure. One of the most common issues is the long waiting list for colonoscopies, affecting 29% of the service. To increase the uptake of CCE services, it is essential to emphasise both the ease of access to CCE and its potential to address the current imbalance between patient demand and service provision.

The survey also highlighted a significant suggestion of using CCE in patients who declined or did not tolerate colonoscopy, leading to incomplete procedures. This aligns with the latest ESGE guideline 2020, which supports using CCE in patients with non-alarm symptoms and when colonoscopy was not possible, as well as FIT-positive patients following an incomplete colonoscopy [11].

Despite the limited evidence for CCE in monitoring and surveillance of inflammatory bowel disease (IBD) outlined in the ESGE guidelines, it remains the second most common indication for its use in our survey. Our findings demonstrate an increasing reliance on CCE for IBD management, even though it lacks the capability for obtaining biopsies. A recent systematic review conducted by Lei et al. reported a pooled accuracy (AUC) of 0.93 for ulcerative colitis and 0.87 for Crohn’s disease [7], confirming the high diagnostic accuracy of CCE in detecting IBD. This trend can also be attributed to CCE’s capability for panenteric examinations, visualising both small and large bowels, like its counterpart, the Crohn’s^TM^ capsule [18,19]. A systematic review and meta-analysis of the panenteric capsule endoscopy (PCE), including seven studies each for ulcerative colitis and Crohn’s disease, showed a sensitivity of 93.8% in UC detection. At the same time, PCE was likely superior in detecting Crohn’s disease compared to colonoscopy and magnetic resonance enterography [7]. However, due to the small number of studies and sample sizes, more research is indicated in using CCE in IBD.

For other relatively common indications, the ScotCap study and NHS England pilot project focused on using CCE in low-risk symptomatic patients and for post-polypectomy surveillance (7% and 8%). Using CCE for lower GI bleeding (7%) might be particularly helpful in the cases of recurrent iron deficiency and/or overt GI bleeding following a non-diagnostic oesophagogastroduodenoscopy [20].

The χ^2^ test for goodness of fit revealed significant differences between various age and fitness categories for CCE usage. Although the specialists’ votes indicated a preference for younger patients, a closer analysis showed that fitness is the most critical deciding factor. Specifically, 77% of specialists expressed a preference for using CCE in physically fit patients, excluding those who are frail or generally struggle with technology.

A large observational study in France by Benech et al. demonstrated that the elderly population was primarily targeted for CCE due to contraindications to anaesthesia or colonoscopy [21]. This study also highlighted a low completion rate and adequate bowel preparation rate of 48.9%. Consequently, there is a tendency toward younger patients, likely due to their better quality of bowel preparation, ability to handle technology, and ability to complete the rigorous bowel preparation and booster regimen. Macleod et al. reported similar findings in the ScotCap study, identifying age as an important predictive factor for a successful CCE test because younger patients may be more capable of completing the complex bowel preparation and booster regimen [21]. Conversely, drawing on research from colonoscopy, a related field, a study by Sachdeva et al. reported no correlation between age and the likelihood of incomplete colonoscopy [22]. Therefore, further investigation is needed to understand if younger age is directly associated with a higher success rate for CCE procedures.

Regarding the perception of CCE among gastroenterologists and lower GI surgeons, only 27% considered it a good alternative, while 24% were sceptical and reluctant to use CCE. Additional challenges included the capsule’s high cost, the procedure’s time-consuming nature, and the difficulty of the preparation process. In total, 57% of the perceptions were negative.

Interestingly, 16% of CCE specialists highlighted a lack of knowledge or awareness about this technology as a potential barrier to its uptake. While this percentage may appear low, its impact is likely to be far more significant, as limited knowledge can perpetuate broader scepticism regarding the utility of CCE. This is particularly evident in the perception among general surgeons and gastroenterologists that the evidence supporting CCE’s diagnostic accuracy remains insufficient despite substantial research. For example, seven meta-analyses on CCE for polyp detection report pooled sensitivities ranging from 79% to 96% and specificities between 86% and 88% [23]. Similarly, the previously cited meta-analysis on CCE for IBD detection demonstrated an overall pooled sensitivity and specificity of 90% and 76%, respectively, with an Area Under the Curve (accuracy) of 0.92 [6].

This relates to the previously discussed points that (a) the lack of national guidelines and (b) the service key performance index (KPI) exacerbate the issue by failing to standardise and disseminate existing evidence on CCE. Such guidelines could be crucial in enhancing awareness and building confidence in its clinical use. Notably, all the invited participants in this study were CCE specialists with a strong understanding of the evidence supporting CCE. As a result, the survey design did not include questions on the evidence base, as it was assumed to be well-understood within this specialist cohort.

In terms of clinicians’ perceived patient perceptions of CCE, 50% viewed it positively. In comparison, 38% had negative views due to factors such as the need for conversion to colonoscopy, long procedure times (with some European countries requiring patients to remain within the unit throughout the procedure), high costs, and intensive bowel preparation. Additionally, 22% of responses were unknown, as CCE is not routinely used. These results are similar to the meta-analysis conducted by Deding et al., which showed a pooled patient preference of 52% for CCE and 45% for colonoscopy, without any statistically significant difference [24].

Bowel preparation regimens can be classified into three categories: PEG with two additional laxatives, PEG with one additional laxative, and PEG alone. The most used regimen is PEG, with two additional laxatives, chosen by 38% of respondents. This preference is due to the critical importance of high-quality bowel cleansing in CCE, given the inability to wash the mucosa as in colonoscopy. A meta-analysis conducted by Bjoersum-Meyer et al. showed that PEG combined with magnesium citrate achieved the highest cleansing rate [25]. However, in this survey, PEG with one additional laxative was not very popular, and no centres reported using magnesium citrate.

Regarding PEG dosage, 49% of respondents used a 2-litre dose, while 34% used a 4-litre dose. The variation in booster regimens was even wider compared to bowel preparation, with 14 different regimens reported. NaP was the most used booster at 23%, followed by PEG at 18%. These findings highlight significant variability in both bowel preparation and booster regimens, indicating a need for an optimal regimen to maximise completion and bowel cleansing rates.

When predicting the future use of CCE, 74% of clinicians expressed a positive outlook, while 6% expressed that their optimism will depend on the funding for CCE and 3% on its price. One reason suggested in the survey for this positive outlook was the potential of CCE for panenteric visualisation, which can help identify and triage patients to the appropriate test, such as colonoscopy or enteroscopy. As the technology advances, particularly with the incorporation of AI, it is hoped that the costs of CCE capsules and services will decrease [26]. High costs remain one of the main hurdles for CCE in alleviating the workload of colonoscopy services. Moreover, various strategies have been proposed to address CCE-to-colonoscopy conversion, including risk stratification with qFIT [6], polyp matching criteria to prevent duplicate reporting [27], and optimising polyp sizing systems in CCE [28].

Even with the predominant academic interest in understanding CCE, the issue of cost-effectiveness remains critical, as it ultimately determines the sustainability of healthcare services. Although the survey did not specifically address individual factors, such as the cost of the capsule, this concern emerged repeatedly in the free-text comments from respondents. According to the Healthcare Improvement Scotland (SHTG) and ScotCap study, the estimated total cost per CCE procedure was £747 (€900) at list price, compared to £900 (€1085) for a colonoscopy based on National Services Scotland micro-costing data [13,23]. Economic analysis from SHTG indicated that CCE increased financial costs within their healthcare system. This was primarily attributed to colonoscopy reinvestigation resulting from poor bowel cleansing, incomplete procedures, and the need for biopsies or interventions for identified pathologies. Despite these challenges, one important potential cost-saving aspect of CCE, even with post-CCE reinvestigations, is the ability to downgrade an urgent colonoscopy to a non-urgent flexible sigmoidoscopy, which is significantly less expensive than a colonoscopy. However, countries that do not utilise flexible sigmoidoscopy in their clinical practice may not be able to take advantage of this potential cost-saving benefit.

In this context, integrating CCE with telemedicine could potentially further reduce costs by enabling service upscaling and incorporating automated systems. Unlike traditional colonoscopy, CCE is not limited by the availability of endoscopy units, scopes, or trained endoscopists, making it a highly scalable diagnostic option. This potential was demonstrated by Nia et al., who leveraged 5G technology and a home-delivery Smartbox system, showcasing the feasibility of remote CCE deployment, particularly its potential to reach underserved communities and improve health inequalities [29].

Based on the survey findings, ensuring the widespread adoption and success of CCE in future clinical practice will likely involve the following strategies:

- Target Patient Group: Focusing on younger and, most importantly, physically fit patients as the primary candidates for CCE.
- Key Indications: Including low-to-moderate risk symptomatic patients, those with incomplete colonoscopy procedures, and individuals with inflammatory bowel disease (IBD) as key indications for CCE.
- Risk Stratification: Utilising advanced risk stratification tools to minimise subsequent colonoscopy conversion rates, thereby improving cost-effectiveness and reducing the overall carbon footprint.
- Cost Reduction: Lowering costs through the potential integration of AI applications and scalability via telemedicine.
- Optimisation of Protocols: Enhancing bowel cleansing and booster regimens to improve diagnostic accuracy, addressing a critical concern for general gastroenterologists and surgeons.

### Limitations

The primary limitation of this study is the small number of respondents, which makes it challenging to draw definitive conclusions and reduces the comprehensiveness of the findings. This limited response may reflect limited exposure to CCE, particularly in European countries that did not participate in the survey. It also suggests that the number of clinicians specialising in CCE across Europe remains relatively small. Moreover, the respondents were likely enthusiastic about CCE technology, and the survey specifically targeted clinicians with a pre-existing interest in CE. These factors introduce selection bias. However, it would equally be challenging to gather representative and meaningful information about CE services in those countries without targeting those with specific interests. Future surveys should consider including colleagues from CE centres who may have different views on the technology to provide a more balanced perspective. This survey should also include the current knowledge or understanding evidence aspect of CCE to understand how much the lack of knowledge is directly link to the poor uptake.

## 5. Conclusions

This study has shown a wide variation in CCE practice and identified some factors contributing to its uptake. Understanding the most common reasons for using CCE and the specific groups of people for whom the procedure is most successful will help guide the future development of this technology. With its potential to visualise the entire digestive tract, enhanced by AI and telemedicine with the cloud-based system, CCE has the potential to help meet the increasing demand for endoscopy services.

## Figures and Tables

**Table 1:** Survey sections and questions.

CAPTURE-EU Questionnaire 2024
Participant Information
Participants demographics
2. Which country are you based in?
Accessibility of Colon Capsule Diagnostics
3. Is CCE being used in your country, and is it readily accessible for your patients?
4. If not, why not? Is it funded?
5. Do you have a CCE service locally, or is it necessary to refer patients elsewhere for a CCE?
6. Are there access issues with other diagnostic tests in your country?
7. Are there access issues with diagnostic tests in your country (CTC/colonoscopy etc)? Do you think CCE could help if funded?
8. Do you currently have access to CT colonography? If so, is colon capsule endoscopy (CCE) preferred over CTC (CT colonography), and if applicable, in which patient groups is it prioritised?
9. Does CCE feature in your national/local guidelines?
10. Are national data for CCE accessible? If affirmative, may we kindly request a copy mainly for data analysis?
CCE Indications and Target Patient Demographics
11. If CCE is in use, in which patient demographics or conditions is it applied?
12. If CCE is in use, which age group would be clinically preferred?
Perceptions of CCE by Clinicians and Patients
13. How is CCE perceived by clinicians (the overall attitude toward CCE from gastroenterologists and surgeons)?
14. How do patients perceive CCE locally?
15. How do you think CCE will be used locally in the future?
Procedural Details of CCE
16. If CCE is used, what bowel preparation regime does your centre use?
17. What bowel preparation dosage does your centre use?
18. If CCE is used, what booster regime does your centre use?
19. We are in the process of compiling information to create an overview of current colon capsule endoscopy (CCE) utilisation across Europe. Do you know anyone with the use of CCE in the following countries: the Republic of Cyprus, Estonia, Finland, Latvia, Lithuania, Luxembourg, Slovakia, and Slovenia?
20. Do you have any suggestions or additional comments for us that may be helpful for this project?

**Table 2:** Summary of the differences in CCE practice in different European countries.

Country	No. of Responses	CCE Accessible	CCE in Use	National Guideline	Number of Indications for CCE	Patient Fitness	Age Range
Denmark	1	Yes	Yes	No	2	Fit & Frail	Age > 18
Italy	8	Yes	Yes	Yes	1	Fit & Frail	Age > 18
Sweden	3	Yes	Yes	No	4	Fit & Frail	Age > 18
Romania	1	Yes	Yes	No	1	Fit & Frail	Age > 18
Netherlands	2	No	Yes	No	3	Fit	Age > 18
England	2	Yes	Yes	Yes	3	Fit	Age > 18
Ireland	1	Yes	Yes	No	7	Fit	Age > 18
France	1	Yes	Yes	Yes	1	Frail	Age > 75
Belgium	1	No	No	No	-	-	
Croatia	1	Yes	No	No	-	-	
Germany	2	Yes	No	No	-	-	
Portugal	3	Yes	Yes	No	3	Fit	18 < Age < 65
Poland	1	Yes	Yes	No	4	Fit	Age > 18
Spain	3	Yes	Yes	Yes	6	Fit & Frail	Age > 18
Turkey	1	Yes	Yes	No	1	Frail	(Age > 75)
Greece	1	Yes	Yes	No	1	-	
Czech Republic	1	Yes	Yes	No	2	Fit	Age > 18
Hungary	1	Yes	Yes	No	3	Fit	Age > 18
Scotland	1	Yes	Yes	Yes	5	Fit	Age > 18

**Figure A2:**
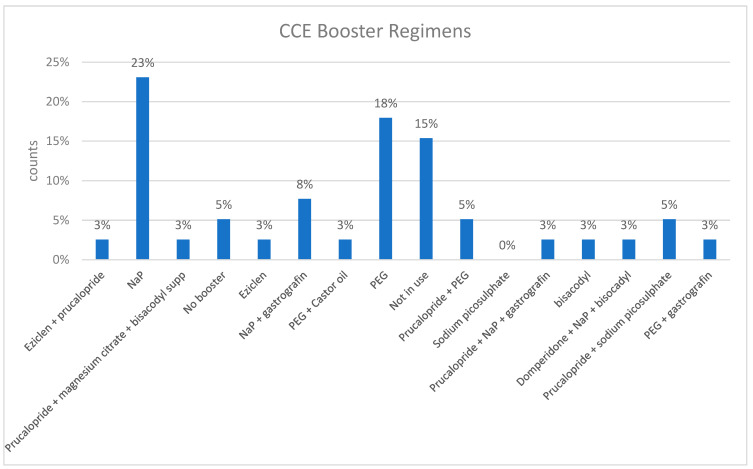
Popularity of different bowel booster regimen.

**Figure A3:**
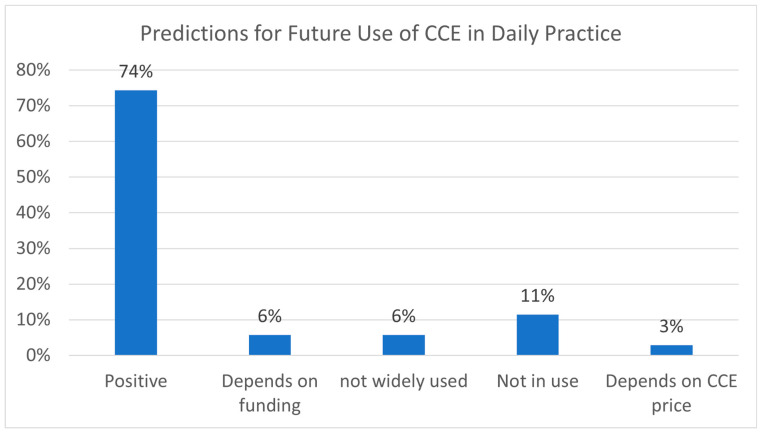
Predictions for future use of CCE in daily practice.

## Data Availability

Dataset available on request from the authors.

## Abbreviation

AI	Artificial intelligence
CCE	Colon capsule endoscopy
CD	Crohn’s disease
COVID-19	Coronavirus disease 2019
CRC	Colorectal cancer
CT	Computed tomography
CTC	Computed tomography colonography
ESGE	European Society of Gastrointestinal Endoscopy
GI	Gastrointestinal
HNPCC	Hereditary non-polyposis colorectal cancer
IBD	Inflammatory bowel disease
IC	Ileocolonoscopy
iCARE group	International Capsule Endoscopy Research Group
MRI	Magnetic resonance imaging
MRE	Magnetic resonance enterography
NaP	Sodium phosphate
NHS	National Health Service
NPV	Negative predicted value
PCE	Panenteric capsule endoscopy
PEG	Polyethylene glycol
qFIT	Quantitative faecal immunochemical test

## Appendix A

**Table A1:** Chi-square test of independence of different factors to identify any statistically significant pattern.

Chi-Square Test of Independence			
Variable 1	Variable 2	χ^2^	*p*-Value
CCE in National guideline	Other GI tests access	1.371	0.242
Other GI tests access	CCEvsCTC preference	1.861	0.173
CCEvsCTC preference	Future CCE usage	0.155	0.694
Other GI tests access	Future CCE usage	2.584	0.108
CCE in hational guideline	Future CCE usage	1.898	0.168
CCE in National guideline	CCEvsCTC preference	0.0182	0.892
Current CCE service availability	CCE in national guideline	1.114	0.292
Current CCE service availability	Other GI tests access	0.501	0.479
Current CCE service availability	CCEvsCTC preference	0.338	0.561
Current CCE service availability	Future CCE usage	2.028	0.154
Current CCE service availability	CTC availability	1.06	0.304
Current CCE service availability	Difficulty in CCE access	3.110	0.375
Current CCE service availability	Challenges/Ease to CCE access	13.5	0.0358 *

* Indicate statistical significance with a *p*-value < 0.05.

**Table A2:** Univariate analysis and multivariate analysis of different factors that might contribute to local CCE service availability.

	Univariate Analysis		Multivariate Analysis	
	Odd Ratio (95% CI)	*p*-Value	Odd Ratio (95% CI)	*p*-Value
Local CCE availability	Reference			
Local CTC availability	5.143 (0.516–51.22)	0.163	5.3 (0.352–79.736)	0.228
National CCE guideline	- (data sparsity)	0.992	- (data sparsity)	0.995
CTC vs. CCE preference	0.490 (0.115–2.09)	0.335	0.596 (0.083–4.261)	0.605
Future CCE use	2.386 (0.558–10.20)	0.241	3.809 (0.575–25.219)	0.166
GI Test Access issues	0.916 (0.243–3.463)	0.898	1.353 (0.215–8.526)	0.167

Binary logistic regression:
R Code used:

1. glm(formula = Availability ~ Nationalguidelines, family = “binomial”, data = Binary_linear_regression)
2. glm(Availability ~ Access, data = Binary_linear_regression, family = “binomial”)
3. glm(formula = Availability ~ CTCvsCCE, family = “binomial”, data = Binary_linear_regression)
4. glm(formula = Availability ~ FutureCCE, family = “binomial”, data = Binary_linear_regression)
5. glm(formula = Availability ~ CTCavailability, family = “binomial”, data = Binary_linear_regression)

Multivariate logistic regression model:
R Code used:

model < -glm(Availability ~ Access + Nationalguidelines + FutureCCE + CTCvsCCE + CTCavailability, data = data, family = binomial)

summary(model)

**Table A3:** The breakdown of various CCE indications across different EU countries.

Country	Number of Indications for CCE	Patient Under the Suspected Cancer Pathway	Patient with Any Lower Gastrointestinal Symptoms	Patient in the Bowel Cancer Screening Programme	Patient Suspected with Inflammatory Bowel Disease	Patient Declined or Did Not Tolerate Colonoscopy (Incomplete Colonoscopy)	Patient Under the Post-Polypectomy Surveillance	Patient with Hereditary Colorectal Conditions	Patient for Post-Colorectal Cancer Resection Surveillance	Patient for IBD Surveillance & IBD Monitoring	Patients with Acute Lower GI Bleed	Family History Screening	Patient Preference	On Anticoagulants or Anti-Platelets	Upper/SB GI Bleed	Not in Use
Denmark	2				✓	✓										
Italy	1					✓										
Sweden	4		✓		✓	✓				✓						
Romania	1												✓			
Netherlands	3		✓		✓	✓										
England	3	✓				✓	✓									
Ireland	7		✓		✓	✓	✓				✓	✓	✓			
France	1					✓										
Belgium	-															✓
Croatia	-															✓
Germany	-															✓
Portugal	3				✓	✓				✓						
Poland	4	✓		✓		✓	✓									
Spain	6				✓	✓		✓		✓	✓			✓		
Turkey	1					✓										
Greece	1					✓										
Czech Republic	2									✓	✓					
Hungary	3					✓			✓	✓						
Scotland	5	✓	✓			✓	✓		✓							
